# Myricetin Nanofibers as Amorphous Delivery System

**DOI:** 10.3390/ph19030449

**Published:** 2026-03-10

**Authors:** Natalia Rosiak, Wojciech Rydyger, Andrzej Miklaszewski, Judyta Cielecka-Piontek

**Affiliations:** 1 Department of Pharmacognosy and Biomaterials, Faculty of Pharmacy, Poznan University of Medical Sciences, 3 Rokietnicka St., 60-806 Poznan, Poland; nrosiak@ump.edu.pl (N.R.); wojciech.rydyger@gmail.com (W.R.); 2 Faculty of Materials Engineering and Technical Physics, Institute of Materials Science and Engineering, Poznan University of Technology, 60-965 Poznan, Poland; andrzej.miklaszewski@put.poznan.pl

**Keywords:** myricetin, nanofiber mat, electrospinning, Box–Behnken

## Abstract

**Background:** Myricetin (MYR) is a natural flavonol with antioxidant, neuroprotective, anti-inflammatory, antidiabetic, and cardioprotective activities. Still, its pharmaceutical use is limited by very low aqueous solubility (~16.6 µg/mL) and poor oral bioavailability (<10%). This study aimed to enhance the solubility and potentially improve the bioavailability of MYR by developing an amorphous nanofibrous delivery system. **Methods:** Electrospinning was applied to fabricate MYR-loaded nanofibers using polyvinylpyrrolidone K30 (PVP30), and the influence of key processing parameters on MYR solubility was evaluated. Nanofibers produced under selected electrospinning conditions were characterized in terms of morphology, encapsulation efficiency, and physicochemical properties. **Results**: X-ray powder diffraction confirmed complete amorphization of MYR within the BB5 fiber structure (distance: 12 cm, voltage: 25 kV, flow rate: 1.5 mL/h). FTIR analysis indicated hydrogen-bonding interactions between MYR hydroxyl groups and PVP30 carbonyl groups, contributing to stabilization of the amorphous form. SEM images revealed homogeneous, defect-free fibers with diameters below 400 nm, although localized MYR agglomerates were observed. Solubility and release studies demonstrated a characteristic spring-and-parachute effect, enabling rapid MYR release and maintenance of a supersaturated state. Enhanced solubility resulted in significantly improved antioxidant activity in DPPH and CUPRAC assays compared with crystalline MYR. **Conclusions:** Electrospun PVP30 nanofibers represent a promising platform for improving the solubility, dissolution behavior, and functional activity of poorly soluble bioactive compounds such as myricetin, supporting their potential application in pharmaceutical formulations.

## 1. Introduction

Polyphenols constitute a broad group of naturally occurring compounds widely distributed in plants, where they play important protective and metabolic roles. Due to their strong antioxidant, anti-inflammatory, and anticancer properties [1,2,3,4], polyphenols have attracted significant attention as bioactive agents with potential applications in pharmaceutical, cosmetic, and food industries [5,6,7]. However, despite their promising biological activities, many polyphenols exhibit unfavorable physicochemical properties, including low aqueous solubility and limited intestinal permeability, which severely restrict their bioavailability and therapeutic effectiveness [8,9,10,11].

Myricetin (3,5,7,3′,4′,5′-hexahydroxyflavone, MYR) is a naturally occurring flavonol found in various fruits, vegetables, tea, and wine. Owing to the presence of six hydroxyl groups, MYR exhibits pronounced antioxidant activity and has been reported to exert anticancer, anti-inflammatory, antidiabetic, neuroprotective, and cardioprotective effects through modulation of multiple signaling pathways. Nevertheless, its clinical application is significantly limited by extremely low water solubility (approximately 16.6 μg/mL) and poor oral bioavailability, reported to be below 10%, which classifies MYR as a poorly soluble and poorly permeable compound [12,13,14].

To overcome these limitations, various formulation strategies have been explored, including cyclodextrin inclusion complexes [15,16,17,18], lipid-based carriers [19,20,21], nanocarriers [22,23], and amorphous solid dispersion [14]. Among these approaches, electrospinning has emerged as a particularly promising technique for fabricating nanofibrous drug delivery systems. Electrospun nanofibers are characterized by a high surface-to-volume ratio, porous structure, and the ability to encapsulate bioactive compounds within a polymeric matrix, thereby enhancing their apparent solubility, stability, and release behavior [24,25].

The development of amorphous solid forms is a widely applied strategy to enhance the apparent solubility and dissolution rate of poorly water-soluble polyphenols [26,27,28,29,30,31,32,33,34,35,36,37,38]. Conventional approaches, such as spray drying, freeze drying, hot-melt extrusion, and solvent evaporation, have been extensively used to produce amorphous dispersions [39,40,41,42,43,44,45]. However, these techniques may present certain limitations, including high thermal or mechanical stress, limited control over morphology, particle aggregation, and challenges in achieving uniform drug distribution.

Electrospinning offers a promising alternative for preparing amorphous systems [46,47,48,49,50,51]. Due to the rapid solvent evaporation and high stretching forces during fiber formation, electrospinning effectively suppresses crystallization and promotes molecular-level dispersion of bioactive compounds within the polymer matrix. The resulting nanofibers exhibit a high surface-area-to-volume ratio, which facilitates rapid dissolution and enhances apparent solubility [52,53,54]. Importantly, electrospun nanofibers offer additional functional advantages over conventional amorphous systems. Their structural versatility enables direct application as standalone delivery systems or integration into multilayer constructs [55,56,57]. Nanofibers can be deposited onto secondary substrates, such as hydrogels, wound dressings, transdermal patches, or contact lenses, thereby expanding their applicability in biomedical and pharmaceutical fields [58,59,60,61,62]. This platform flexibility allows simultaneous modulation of drug release kinetics, mechanical properties, and biological performance. Therefore, electrospinning serves not only as a method for amorphization but also as a multifunctional platform technology for the development of advanced delivery systems for polyphenols and other poorly soluble bioactive compounds.

Therefore, this study aimed to develop electrospun polymeric nanofibers loaded with myricetin and to investigate the influence of selected electrospinning parameters using a Box–Behnken experimental design. The study focused on evaluating the effects of process variables on the dissolution performance of MYR, with the goal of enhancing its apparent solubility and antioxidant activity. Based on the results, the selected formulation, meeting predefined dissolution and supersaturation criteria, was subjected to further characterization. The selected nanofibers were thoroughly characterized for morphology and physicochemical properties using scanning electron microscopy (SEM), X-ray powder diffraction (XRPD), and Fourier-transform infrared spectroscopy (FT-IR), and their antioxidant activity was assessed using the CUPRAC and DPPH assays.

## 2. Results and Discussion

### 2.1. Determination of Specific Viscosity

The rheological properties of polymer solutions, particularly viscosity, are crucial to the effective application of polymers in electrospinning. According to the literature [63,64], an appropriate solution viscosity reflects the degree of polymer chain entanglement. This phenomenon occurs only after a critical concentration threshold is exceeded and is essential for maintaining the polymer jet’s continuity during fiber formation. Exceeding this threshold results in a pronounced increase in viscosity, stabilizing the electrospinning process and enabling the production of nanofibers with suitable morphology [65,66,67,68].

**Figure 1:**
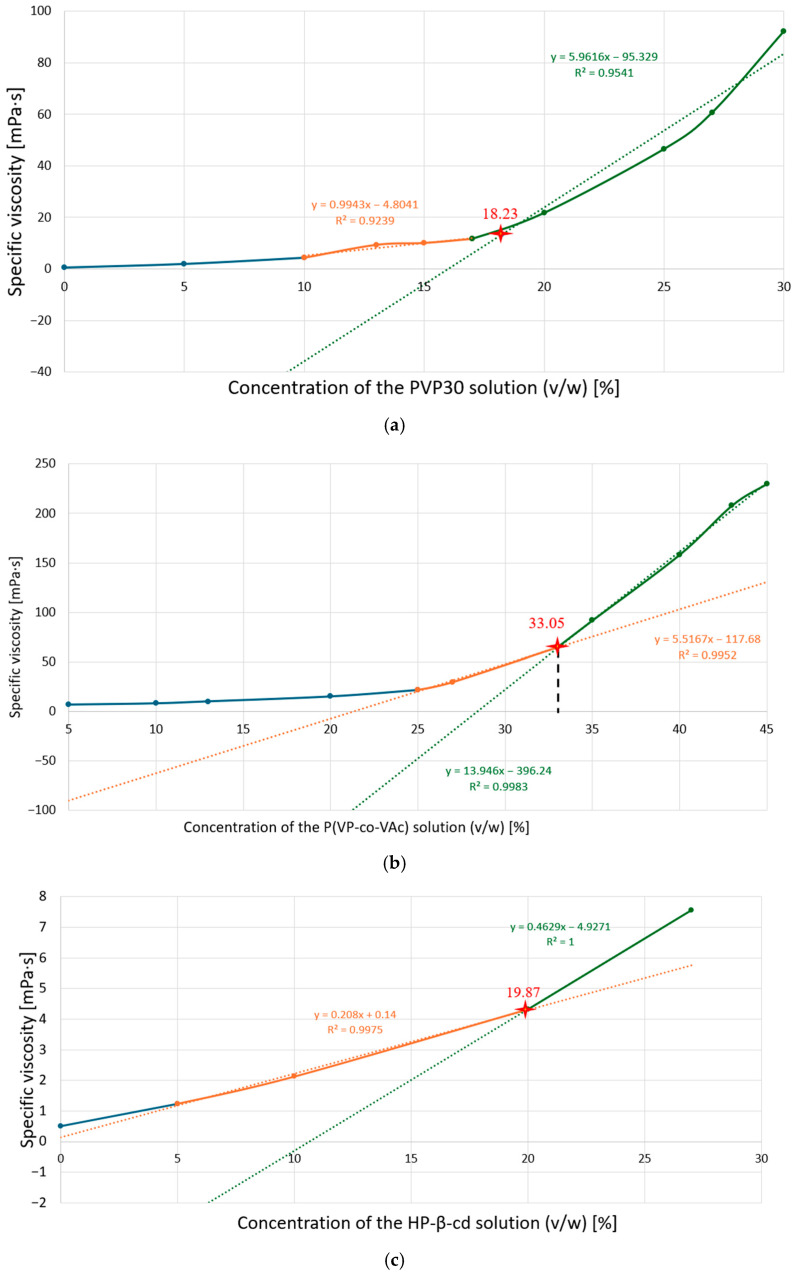
The relationship between specific viscosity and solution concentrations (*w*/*v*) for (**a**) PVP30, (**b**) P(VP-co-VAc), (**c**) HP-β-CD. Red asterisk—the intersection point of the orange and green graphs.

Based on the experimental data, plots of specific viscosity versus polymer concentration were constructed. These plots allowed the determination of the minimum concentration required for the onset of polymer chain entanglement, indicated by the inflection point in Figure 1.

It was indicated that polymer solutions of PVP30, P(VP-co-VAc), and HP-β-CD should be prepared at concentrations of 18% (*w*/*v*), 33% (*w*/*v*), and 20% (*w*/*v*), respectively. Subsequently, the qualification criteria for the polymers to be used in further studies were evaluated. For each polymer, eight electrospinning experiments were performed. Among the tested systems, only the PVP30 solution maintained a stable Taylor cone and a continuous jet without nozzle clogging for at least 60 min. Consequently, PVP30 was selected as the polymeric carrier for further studies and used as the basis for nanofiber fabrication.

### 2.2. Evaluation of Electrospinning Parameters Using a Box–Behnken Experimental Design

Based on the Box–Behnken design matrix, 15 electrospinning experiments were performed. The results of the apparent aqueous solubility studies obtained at time points T_1_ = 15 min and T_2_ = 1 h are summarized in Table 1. It should be emphasized that the term “apparent solubility” used throughout this study refers to the total drug concentration measured in the filtered dissolution medium under the applied experimental conditions. These values do not necessarily represent the true thermodynamic molecular solubility of MYR. Due to the amorphous nature of the system and the presence of polymeric excipients, the measured concentrations may include contributions from supersaturated molecular species, polymer–drug complexes, and/or colloidal or nano-aggregated forms.

Lin et al. [69] reported that MYR:HPβCD:PVP (*w*/*w*/*w*) nanofibers prepared at ratios of 1:20:4, 1:20:8, and 1:20:12 exhibited water solubility values of 0.85 ± 0.02, 0.89 ± 0.01, and 0.91 ± 0.01 mg/mL, respectively. In contrast, the BB1–BB15 developed in the present study demonstrated substantially higher MYR concentrations after 60 min, ranging from 1.73 to 3.06 mg/mL depending on the composition. This corresponds to an approximately two- to threefold increase in apparent solubility compared to the literature data. These results indicate that the developed nanofibrous systems provide a markedly enhanced solubilization effect, which may be attributed to improved amorphization, more favorable MYR–polymer interactions, and increased surface area, which facilitates rapid dissolution.

**Figure 2:**
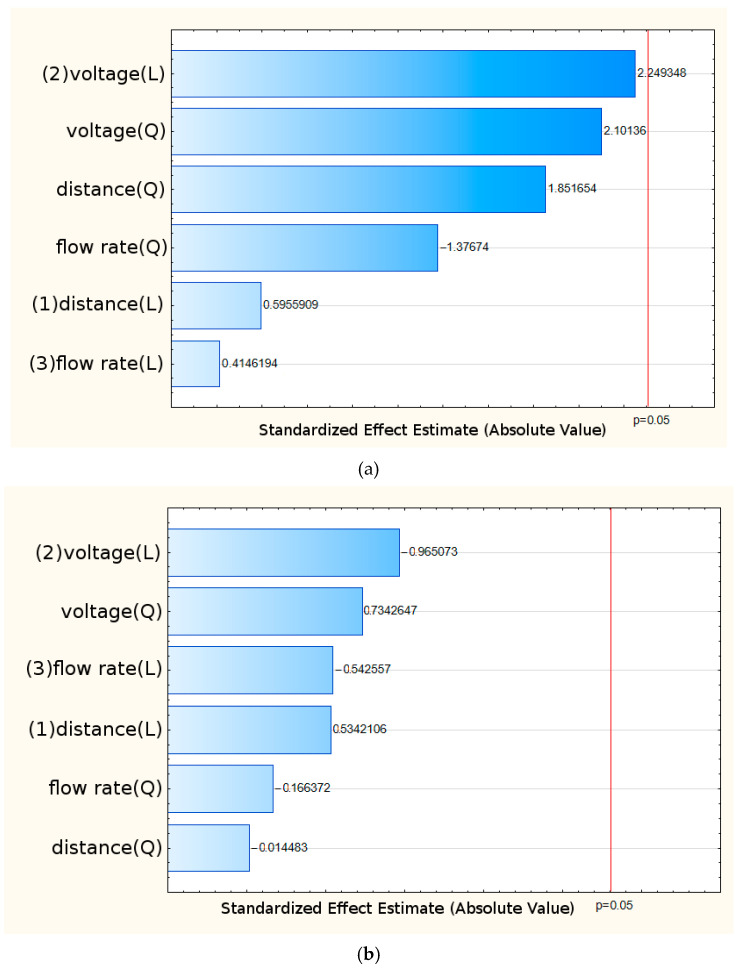
Pareto Chart of standardized effects: (**a**) $T_{1}$ = 15 min and (**b**) $T_{2}$ = 1 h.

The influence of electrospinning parameters on MYR solubility was evaluated by analyzing standardized effects in Pareto charts at two time points: T_1_ = 15 min and T_2_ = 1 h. Included both linear (L) and quadratic (Q) effects of the three investigated variables: applied voltage, solution flow rate, and needle-to-collector distance (Figure 2).

At both 15 min and 1 h of incubation, none of the analyzed effects exceeded the statistical significance threshold (*p* = 0.05), as indicated by the red reference line in the Pareto charts. This suggests that, within the investigated range of electrospinning parameters, no unequivocally significant influence of these factors on MYR solubility was observed. In both cases, the highest standardized effect values were associated with the applied voltage, for both its linear and quadratic components. Although these effects were the most pronounced among the evaluated factors, they did not reach statistical significance. The remaining parameters, namely solution flow rate and needle-to-collector distance, also did not significantly affect MYR solubility. The lack of statistically significant differences may be attributed to several factors. One possible explanation is that the range of electrospinning parameters examined was too narrow to induce a clear effect on the physicochemical properties of the resulting structures. In addition, MYR solubility may be influenced by other factors, such as formulation composition, polymer type, or intermolecular interactions, which were not directly analyzed in this part of the study. Nevertheless, the preliminary trend indicating a relatively greater contribution of voltage suggests that this parameter may warrant more detailed optimization in future studies, either by expanding the tested range or by considering its interactions with other process variables. Due to the inconclusive results regarding the influence of electrospinning parameters on MYR solubility, a more in-depth evaluation of selected systems was undertaken. In particular, attention was focused on their ability to maintain a supersaturated state after 24 h of incubation, a critical criterion for enhancing the bioavailability of poorly soluble compounds. The corresponding results are summarized in Table S1.

The Box–Behnken design for samples after 24 h did not reveal statistically significant effects of the studied variables on the analyzed response parameters, as confirmed by the Pareto charts (*p* > 0.05). Therefore, no statistically substantial optimization effect was observed within the tested design space. Consequently, BB5 (distance: 12 cm, voltage: 25 kV, flow rate: 1.5 mL/h) was selected for further studies based on predefined practical performance criteria rather than statistical optimization. This formulation exhibited a high MYR concentration after 24 h and maintained a stable supersaturated state, with no noticeable decline in concentration over time. Since prolonged supersaturation is considered an important parameter influencing potential bioavailability enhancement, this behavior served as the basis for selecting the formulation for subsequent investigations.

TTo evaluate the reproducibility and reliability of the electrospinning process, three independent batches of fibers were prepared under conditions corresponding to experiment BB5, labeled A, B, and C. For each batch, fibers were collected from three randomly selected locations (designated as 1, 2, and 3), and the actual concentration, predicted concentration, and encapsulation efficiency (EE) of MYR in the nanofibers were determined. The results are summarized in Table 2.

Encapsulation efficiency (DEE) represents a critical parameter in the development of an effective nano-scale active compound delivery system. The mean MYR loading efficiency was 68.96% ± 12.84% for BB5-A, 66.60% ± 3.20% for BB5-B, and 79.33% ± 3.24% for BB5-C. Statistical analysis revealed no significant differences among the analyzed formulations (*p* > 0.05), indicating comparable incorporation of MYR within the developed nanofibrous systems.

Importantly, the obtained encapsulation efficiencies are comparable to or slightly lower than those reported for other polyphenol-loaded nanofibers. For example, curcumin-loaded zein and gelatin fibers demonstrated encapsulation efficiencies in the range of 86–89% [70]. Similarly, encapsulation efficiencies of 83% for curcumin-neem/PCL nanofibers and 77% for curcumin-aloe vera/PCL systems [71]. Moreover, Rostami et al. [72] reported an encapsulation efficiency of 86 ± 6% for resveratrol-loaded chitosan:gellan nanofibers. These results confirm that the encapsulation efficiency achieved in the present study falls within the typical range reported for bioactive-loaded electrospun nanofibers and demonstrates effective incorporation of MYR into the developed polymer matrices.

### 2.3. Nanofiber Characterization

#### 2.3.1. Scanning Electron Microscopy (SEM) Analysis

SEM is widely used to evaluate nanofiber morphology, diameter distribution, and surface structure. Owing to its high resolution, SEM enables detailed assessment of fiber uniformity, the presence of defects (e.g., bead formation), and the spatial orientation of fibrous mats. Due to its precision and versatility, SEM remains one of the most important techniques for characterizing nanostructured materials, including electrospun fibers used in tissue engineering, drug delivery systems, and filtration materials [50,73].

SEM analysis was employed to evaluate the morphology of the nanofibrous mats, with particular emphasis on structural uniformity, fiber diameters, surface defects, and the influence of MYR incorporation on the electrospinning process. A control mat based solely on PVP30 and three MYR-loaded BB5 fiber series (A–C) produced under identical processing parameters were analyzed to assess process reproducibility. A summary of fiber-diameter measurements from SEM analysis is presented in Table 3.

**Figure 3:**
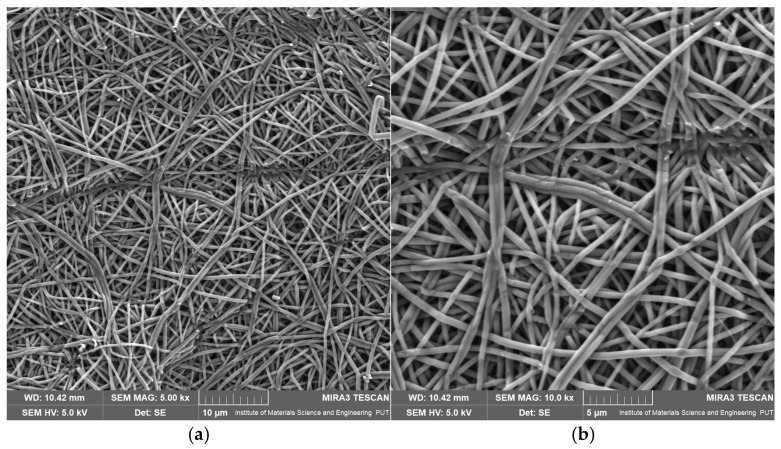
SEM images of electrospun nanofibrous mats: (**a**) PVP30 at 5000× magnification, (**b**) PVP30 at 10,000× magnification.

The control PVP30 mat, without MYR incorporation, showed low mean fiber diameters of 0.755 µm and 0.558 µm, with moderate variability (Figure S1). SEM micrographs (Figure 3a,b) reveal a fibrous structure with a very high degree of uniformity and reproducibility.

The fibers are thin, evenly stretched, and well separated, with no visible surface defects, bead formation, or signs of fiber fusion. No agglomerates or disturbances in the electrospinning process were observed. The uniformity of fiber diameters and the regular fiber arrangement confirm that the PVP30 solution concentration (18% *w*/*v*), selected based on intrinsic viscosity analysis, was appropriate for producing high-quality nanofibrous structures. The PVP30 sample served as a reference for further study of MYR-loaded samples.

**Figure 4:**
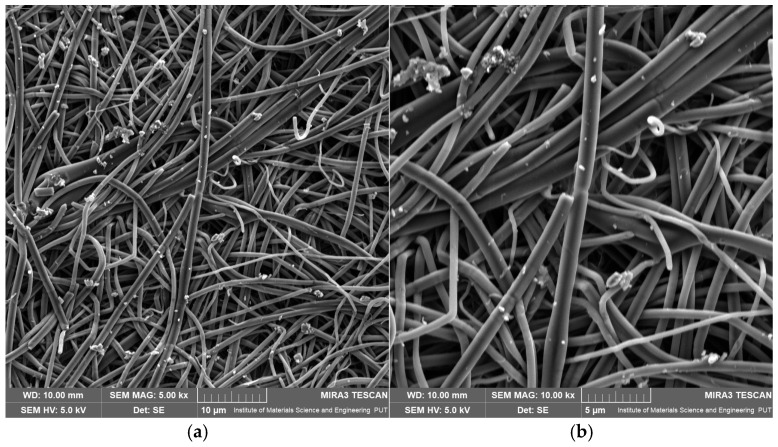
SEM images of electrospun nanofibrous mats: (**a**) BB5-A at 5000× magnification, (**b**) BB5-A at 10,000× magnification.

For BB5-A, the mean fiber diameters ranged from 0.792 to 1.014 µm, with a maximum measured value of 2.958 µm (Figure S2). The observed moderate diameter dispersion may indicate local fluctuations in the electrospinning jet’s stability. SEM images (Figure 4a,b) show that BB5-A fibers are continuous and largely homogeneous, with smooth surfaces.

At the same time, numerous small, irregularly shaped structures, significantly smaller than the fibers themselves, are visible in the immediate vicinity of the fibers. As such objects were not observed in the PVP30 control mat, they can be attributed to MYR aggregates. Their presence suggests incomplete dissolution of the active compound in the methanolic polymer solution or secondary agglomeration during electrospinning. The particle distribution indicates partial precipitation of MYR on the surface of the forming fibers.

**Figure 5:**
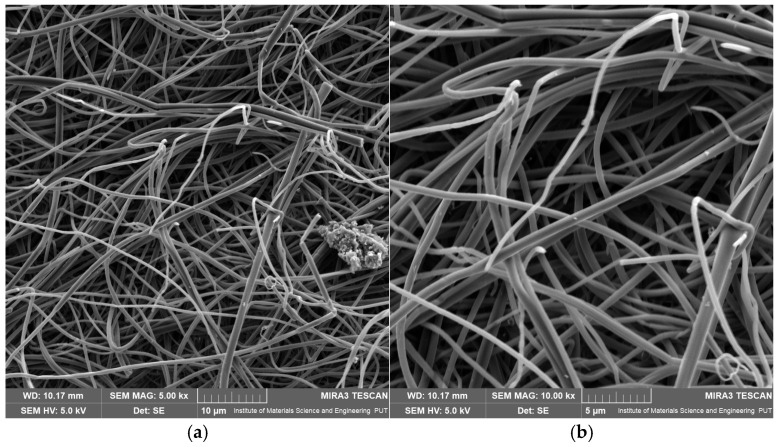
SEM images of electrospun nanofibrous mats: (**a**) BB5-B at 5000× magnification, (**b**) BB5-B at 10,000× magnification.

BB5-B fibers exhibited clearly lower diameters, ranging from 0.614 to 0.723 µm (Figure S3). At the same time, the standard deviations were smaller than those observed for BB5-A, indicating greater structural uniformity. The maximum measured diameter was 1.599 µm, while the minimum was 0.424 µm. SEM micrographs (Figure 5a,b) reveal a fibrous structure with relatively good homogeneity.

The fibers are thin, well-separated, and elongated, a characteristic of a stable electrospinning process. Compared to BB5-A, fewer small agglomerates are observed; however, a single larger material cluster was detected, which may indicate a local excess of MYR or its uneven distribution within the polymer matrix.

**Figure 6:**
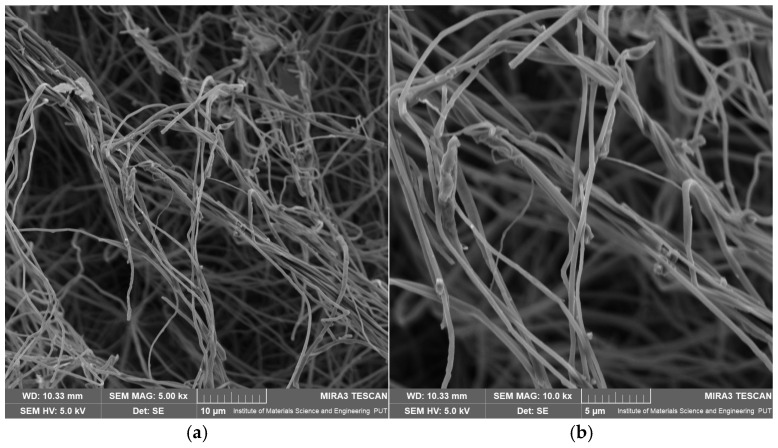
SEM images of electrospun nanofibrous mats: (**a**) BB5-C at 5000× magnification, and (**b**) BB5-C at 10,000× magnification.

For BB5-C, mean fiber diameters of 0.580 µm and 0.505 µm were obtained, accompanied by low standard deviations (Figure S4). Despite these favorable statistical values, SEM images (Figure 6a,b) reveal pronounced differences in morphological quality compared to the other series.

BB5-C fibers show greater local structural heterogeneity, excessive entanglement, and the presence of numerous agglomerates of the active compound. In many regions, irregular fiber trajectories and local densification are observed, which may reduce the mat’s porosity. Such defects may result from non-uniform dissolution of MYR or its secondary agglomeration during electrospinning.

Overall, the results indicate that MYR in the polymer solution affects fiber morphology, leading to increased diameter variability and the formation of active compound agglomerates. Despite identical electrospinning parameters, differences observed among BB5-A, BB5-B, and BB5-C highlight the critical importance of solution homogeneity and sample preparation for achieving reproducible nanofibrous structures.

#### 2.3.2. X-Ray Powder Diffraction (XRPD) Analysis

XRPD is a well-established analytical technique used to investigate the crystalline structure of materials. The method is based on measuring the angles and intensities of X-rays scattered by the ordered crystal lattice of a substance. X-ray diffraction occurs in accordance with Bragg’s law, and the resulting diffraction pattern (diffractogram) is characteristic of a given compound in a specific solid-state form. Amorphous materials, which lack long-range structural order, do not produce sharp diffraction peaks; instead, they exhibit a broad diffuse halo. Consequently, XRPD enables clear differentiation between crystalline and amorphous phases and allows for the assessment of the degree of crystallinity of the analyzed samples [74,75,76,77].

**Figure 7:**
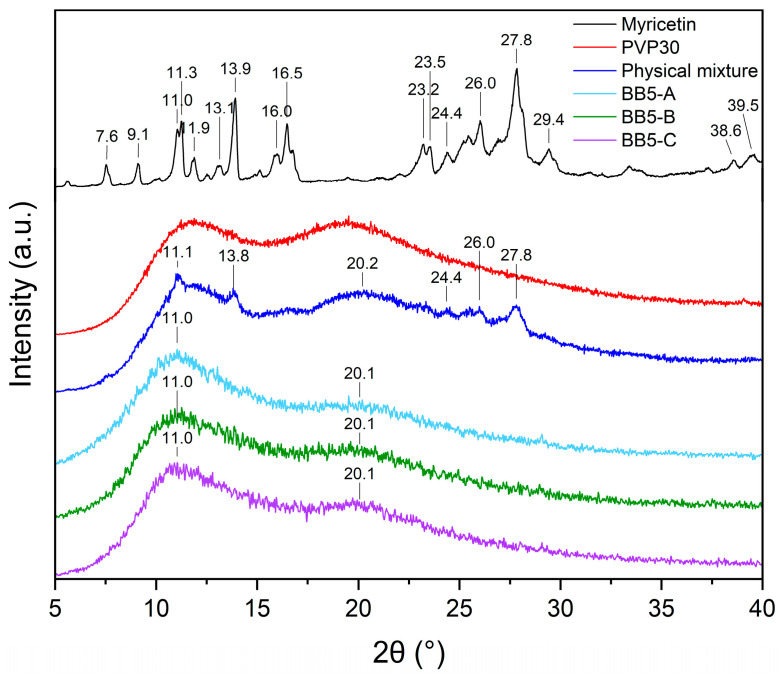
XRPD analysis: X-ray diffraction patterns of MYR, PVP30, the physical mixture of MYR–PVP30, and BB5 nanofibers (A–C).

The XRPD pattern of pure MYR (Figure 7) exhibits numerous sharp and intense diffraction peaks in the 2θ range of 7–30°, with the most characteristic reflections observed at 2θ values of 7.6°, 9.1°, 11.0°, 11.3°, 13.1°, 13.9°, 16.0°, 16.5°, 23.2°, 24.4°, 26.0°, and 27.8°. The presence of these well-defined peaks unequivocally confirms the highly crystalline nature of MYR [14].

In contrast, the XRPD pattern of PVP30 shows no sharp diffraction maxima. Instead, a broad diffuse halo consisting of two overlapping maxima in the 2θ range of 11–25° is observed, which is characteristic of amorphous materials [14,78].

The diffractogram of the physical mixture of MYR and PVP30 represents a superposition of the amorphous scattering pattern of PVP30 and a limited number of Bragg reflections characteristic of crystalline MYR. Due to the high polymer content (approximately 91%), the characteristic diffraction peaks of MYR are largely masked by the broad amorphous halo of PVP30. On this basis, it can be concluded that simple mechanical mixing of the components did not induce amorphization of MYR. In contrast, pronounced structural changes are observed for the electrospun BB5 fibers. Their XRPD patterns do not exhibit any characteristic Bragg peaks corresponding to crystalline MYR. Instead, a broad diffuse halo in the 2θ range of 10–25° is present, clearly indicating complete amorphization of MYR within the PVP matrix. Although SEM analysis revealed the presence of localized agglomerates within the fibrous mats, no corresponding crystalline reflections were detected by XRPD. This discrepancy can be attributed to the fundamentally different nature of both techniques. XRPD is sensitive to long-range crystalline order and provides bulk-averaged information, whereas SEM reveals local morphological features. The observed agglomerates may therefore consist of amorphous or nanocrystalline MYR domains below the XRPD detection limit, further masked by the dominant amorphous halo of PVP30. Consequently, the absence of MYR Bragg peaks in the XRPD patterns does not exclude the presence of MYR-rich domains observed by SEM.

#### 2.3.3. TG and DSC Analysis

**Figure 8:**
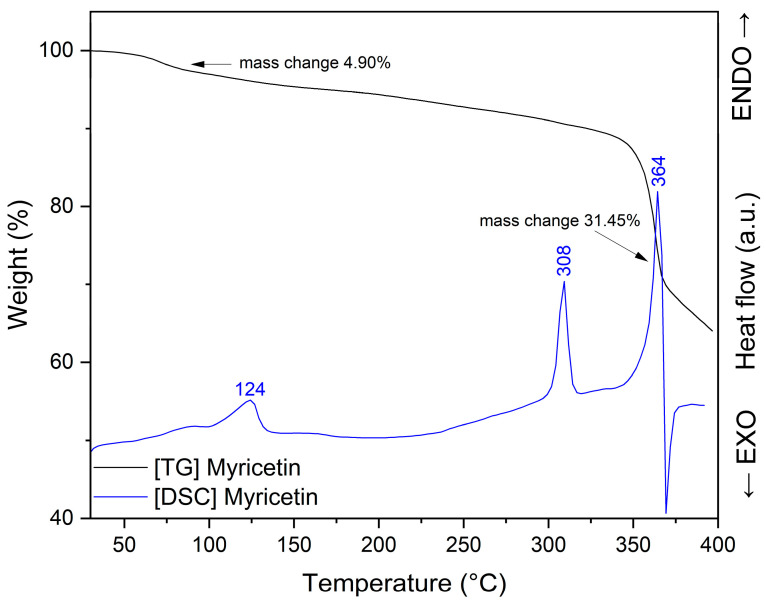
TG and DSC analysis: myricetin.

Thermogravimetric analysis (TGA) was conducted to evaluate the mass loss of myricetin as a function of temperature and to assess the presence of physically bound or crystalline water in the sample. The TG curve (Figure 8) shows an initial mass decrease occurring below approximately 150 °C, with two distinguishable stages that correspond well to the thermal events observed in the DSC profile (around 86 °C and 124 °C). The total mass reduction in this temperature region is approximately 4.90%, attributed to sample dehydration. This weight loss indicates the release of water molecules bound to myricetin’s crystal structure.

At higher temperatures, a more pronounced mass loss is observed, beginning above ~250–300 °C, with a significant decomposition step centered near 308 °C, followed by a major thermal event around 365–370 °C, consistent with the melting and subsequent degradation seen in the DSC curve. The main decomposition stage is characterized by approximately 31.45% mass loss.

Differential Scanning Calorimetry (DSC) is a widely used thermal analysis technique for investigating the thermal behavior and phase transitions of materials [79]. The method measures the difference in heat flow between a sample and an inert reference as a function of temperature or time under controlled heating or cooling conditions. Thermal events such as melting, crystallization, glass transition, polymorphic transformations, and decomposition are detected as endothermic or exothermic signals on the thermogram [80,81,82]. DSC complements XRPD analysis by confirming the solid-state form and providing insight into the thermal stability and phase behavior of the investigated samples [83,84]. Therefore, it was selected as a complementary technique to confirm the amorphous form of MYR in the obtained nanofibers.

**Figure 9:**
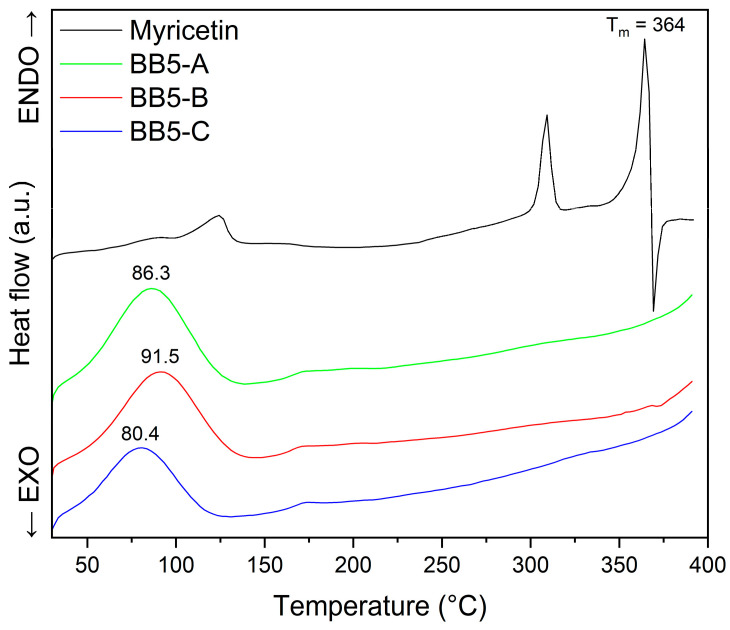
DSC analysis: myricetin, BB5-A, BB5-B, BB5-C.

The DSC thermogram of pure MYR (Figure 9) exhibits a sharp and intense endothermic peak at approximately 364 °C, corresponding to its melting temperature (T_m_) and confirming its crystalline nature [85,86]. Solvent loss (dehydration) is represented by two endotherm around 86 °C and 124 °C. Franklin et al. [86] suggest that endotherm observed at 308 °C is transition of a metastable anhydrous form.

The narrow and well-defined character of this peak indicates a highly ordered crystal structure. In contrast, the DSC profiles of the MYR–PVP nanofibers (BB5-A, BB5-B, and BB5-C) differ markedly from that of the pure compound. In all nanofibers, the characteristic melting peak of MYR at 364 °C is absent, indicating that MYR is no longer present in its crystalline form but is molecularly dispersed within the PVP matrix in an amorphous state. The disappearance of the melting endotherm confirms the successful incorporation of MYR into the polymeric carrier and suggests the presence of intermolecular interactions between MYR and PVP, which inhibit recrystallization. Moreover, the nanofiber samples display broad endothermic events in the temperature range of approximately 80–92 °C (86.3 °C for BB5-A, 91.5 °C for BB5-B, and 80.4 °C for BB5-C), which can be attributed to the evaporation of residual moisture. Overall, DSC results confirm the crystalline structure of pure MYR and demonstrate that, in the MYR–PVP nanofibers, the active compound is present in an amorphous, molecularly dispersed form, as previously confirmed by XRPD analysis.

#### 2.3.4. FT-IR Analysis

**Figure 10:**
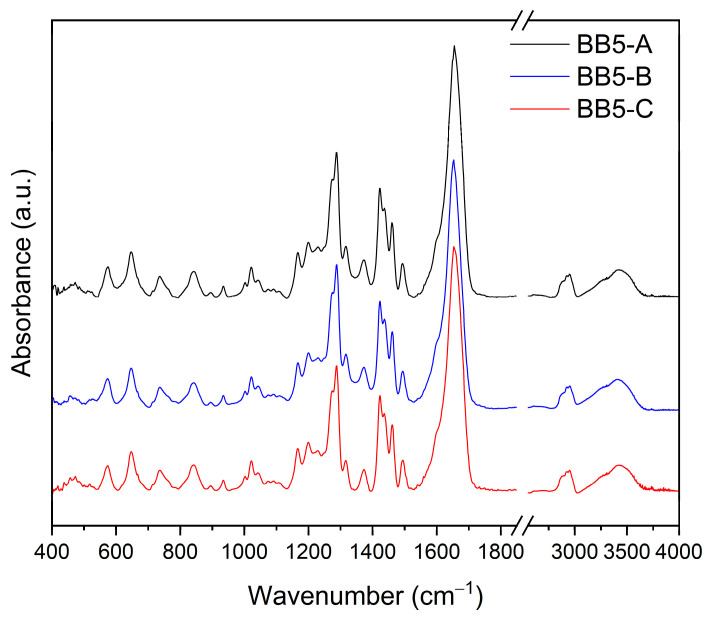
FT-IR analysis: BB5 A–C nanofibers.

To better understand the possible interactions between MYR and PVP30, FT-IR analysis was performed. The recorded spectra for BB5 A–C are presented in Figure 10.

**Figure 11:**
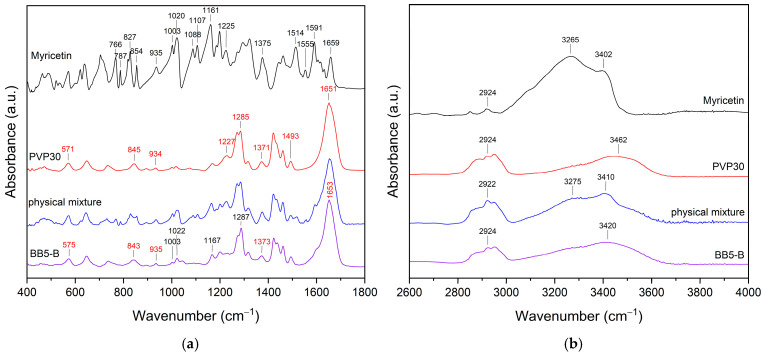
FT-IR analysis: (**a**) MYR, PVP30, the physical mixture of MYR–PVP30, and (**b**) BB5 A–C nanofibers.

Due to the absence of significant differences in the spectral features, BB5-B fibers were selected as a representative sample for further analysis. The FT-IR spectra of MYR, PVP30, the physical mixture of MYR–PVP30, and BB5-B are presented in Figure 11.

The FTIR spectrum of MYR exhibits multiple characteristic absorption bands in the range of 400–1800 cm^−1^, corresponding to vibrations of aromatic rings, carbonyl groups, and hydroxyl functionalities. The bands observed at 1514 and 1591 cm^−1^ are attributed to the stretching vibrations of the aromatic ring, while the absorption at 1659 cm^−1^ is associated with the carbonyl group [87]. In addition, distinct bands are present in the 3200–3600 cm^−1^ region, corresponding to O–H stretching vibrations, with maxima at 3265 and 3402 cm^−1^ [14]. The FTIR spectrum of PVP30 shows a characteristic carbonyl stretching band at 1651 cm^−1^, along with a broad absorption band in the range of 3200–3500 cm^−1^, which is attributed to bound water [14,78]. In the spectrum of the physical mixture, most of the characteristic bands of both MYR and PVP30 are preserved, indicating the absence of strong intermolecular interactions between the components, which is typical for simple physical blending. Changes are observed in the FTIR spectrum of the BB5-B fibers, including band shifts and broadening. Notably, the carbonyl stretching band of PVP30 shifts from 1651 cm^−1^ to 1653 cm^−1^. According to the literature, this functional group participates in hydrogen bonding in amorphous systems [14,78]. Additionally, significant changes are observed in the 3450–3200 cm^−1^ region associated with the O–H stretching vibrations of MYR. While the band at 3265 cm^−1^ characteristic of MYR is still present in the physical mixture, the BB5-B spectrum exhibits only a broad band with a maximum at approximately 3420 cm^−1^. This corresponds to a shift of the 3402 cm^−1^ band by about 18 cm^−1^.

These spectral changes strongly suggest the formation of hydrogen bonds between the hydroxyl groups of MYR and the carbonyl groups of PVP30, confirming molecular-level interactions within the electrospun BB5-B fibers.

### 2.4. In Vitro Supersaturation and Apparent Solubility Study

**Figure 12:**
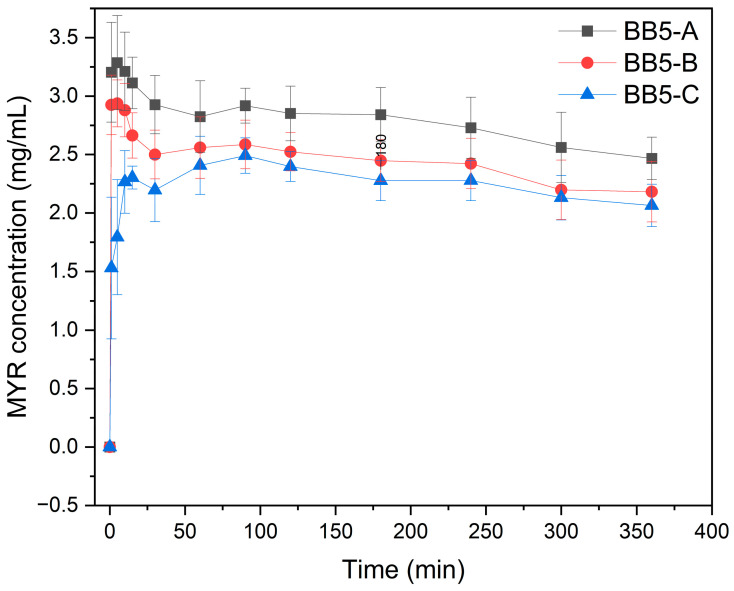
In Vitro Dissolution Test: BB5-A, BB5-B, BB5-C.

The in vitro experiments were conducted under non-sink conditions to evaluate the apparent solubility and supersaturation behavior of amorphous MYR dispersed within PVP30 nanofibers (BB5-A, BB5-B, and BB5-C). The resulting concentration-time profiles are presented in Figure 12.

The BB5 (A–C) nanofibers exhibited a characteristic concentration-time profile typical of amorphous drug delivery systems containing polymeric precipitation inhibitors. This behavior is commonly described as the “spring and parachute effect” [88,89]. The initial rapid increase in MYR concentration (spring phase) reflects the fast generation of supersaturation due to the high apparent solubility of the amorphous form. This was particularly evident for BB5-A and BB5-B, which reached C_max_ values of 3.29 and 2.94 mg/mL, respectively, within 5 min. In contrast, BB5-C showed slower supersaturation build-up, reaching C_max_ (2.49 mg/mL) at 90 min. Importantly, the elevated concentration values observed during the supersaturation phase should not be interpreted as an increase in true molecular solubility. In amorphous solid dispersions, the measured drug concentration may reflect a metastable supersaturated state and can include molecularly dissolved drug, polymer-associated species, and/or colloidal drug-rich aggregates. Therefore, the reported concentrations represent apparent solubility under non-equilibrium conditions rather than thermodynamic equilibrium solubility.

A detailed description of this phenomenon has been provided by Ditzinger et al. [90], who modeled dissolution profiles of amorphous systems and emphasized that the maintenance of the parachute phase depends on stabilizing polymers, dissolution medium properties, and precipitation kinetics.

The initial rapid increase in MYR concentration (spring phase) reflects rapid supersaturation driven by the high apparent solubility of the amorphous form. In the present study, BB5-A and BB5-B reached Cmax values of 3.29 and 2.94 mg/mL, respectively, within 5 min, whereas BB5-C exhibited a slower increase, achieving Cmax (2.49 mg/mL) at 90 min. Following the peak, a gradual decrease in concentration was observed (between 10 and 30 min for BB5-A and BB5-B), corresponding to partial precipitation or molecular reorganization processes. This behavior indicates that MYR remains supersaturated for a defined period. In the present study, the plateau persisted from 100 min until the end of the dissolution experiment (360 min). Ditzinger et al. [90] emphasize that the effectiveness of the parachute effect depends on several factors, including the presence of stabilizing polymers (e.g., PVP, HPMC), the nature of the dissolution medium, the precipitation rate, and the kinetics of crystal nucleation and growth. Hancock et al. [91] demonstrated that amorphous forms exhibit higher thermodynamic solubility and can achieve supersaturation in dissolution media; however, stabilization is required to prevent rapid recrystallization [45]. In addition, review by Baghel et al. [88] confirm the crucial role of polymers as crystallization inhibitors and modifiers of system viscosity.

In the case of the BB5 mats investigated in this study, the nanofibrous structure and the properties of the polymer used most likely contribute to the observed prolongation of the supersaturated state. Differences between BB5-A, BB5-B, and BB5-C may be attributed to variations in fiber microstructure as well as the presence of agglomerates, as confirmed by SEM analysis. Consistent with the dissolution profile results, the BB5-A sample showed the highest MYR release from the PVP30 matrix. These findings correlate well with the observed morphological features: thin, uniform nanofibers facilitate penetration of the dissolution medium into the fibrous mat, thereby enhancing drug release through both diffusion-controlled mechanisms and surface erosion.

### 2.5. Antioxidant Activity

The literature indicates that improving apparent solubility may enhance the compound’s biological properties. MYR exhibits scavenging activity against a wide range of radicals and ions. Numerous studies have reported its antioxidant properties, leaving little doubt that MYR is a potent antioxidant compound [14,19,92,93,94,95].

In the present study, the antioxidant activity was evaluated using two complementary assays: DPPH and CUPRAC. Due to its extremely low aqueous solubility, crystalline MYR did not exhibit measurable antioxidant activity. A pronounced increase in antioxidant activity was observed for the aqueous BB5 solution. In the DPPH assay, the IC_50_ value—defined as the concentration of antioxidant required to inhibit 50% of DPPH radicals—was determined to be 27 ± 2 μg/mL. For comparison, ascorbic acid, used as a reference antioxidant, exhibited a markedly lower IC_50_ value of 9.6 ± 1.2 μg/mL, confirming its stronger radical scavenging capacity. Although BB5 demonstrated lower potency than ascorbic acid (approximately threefold), it showed a pronounced improvement in antioxidant activity compared to pure MYR in aqueous solution, for which no measurable activity was detected due to its limited solubility. When compared with literature data, BB5 exhibited stronger antioxidant activity than an amorphous MYR–PVP dispersion at a 1:9 (*w*/*w*) ratio [14] (IC_50_ = 37.52 ± 3.69 μg/mL), while its activity was approximately two-fold lower than that reported for MYR–PVP120–cyclodextrin nanofibers (IC_50_ = 9.61 ± 0.10 μg/mL) [69].

The CUPRAC assay measures antioxidant capacity by measuring the reducing power of the tested substance toward copper(II) ions (Cu^2+^). The CUPRAC result reflects a sample’s ability to donate electrons and reduce copper ions, which is relevant to the neutralization of reactive oxygen species in biological systems. Consequently, this assay provides complementary information to that obtained from the DPPH test, enabling a more comprehensive assessment of MYR’s antioxidant properties. In the CUPRAC assay, the IC_0.5_ value was determined to be 120 ± 3 μg/mL. For comparison, ascorbic acid exhibited a substantially lower IC_0.5_ value of 10.9 ± 0.8 µg/mL, confirming its markedly higher reducing capacity. In contrast, pure crystalline MYR in aqueous solution did not exhibit measurable antioxidant activity, likely due to its extremely limited solubility and poor water solubility. Activity of BB5 is significantly lower than that reported for the amorphous MYR–PVP 1:9 (*w*/*w*) dispersion (IC_0.5_ = 20.47 ± 9 μg/mL) [14]. To date, only studies on amorphous MYR–PVP dispersions have reported antioxidant activity of MYR-containing systems evaluated using the CUPRAC assay. The lack of additional literature data limits direct comparisons; however, it simultaneously highlights the novelty and scientific relevance of the present study.

### 2.6. Stability Study

**Figure 13:**
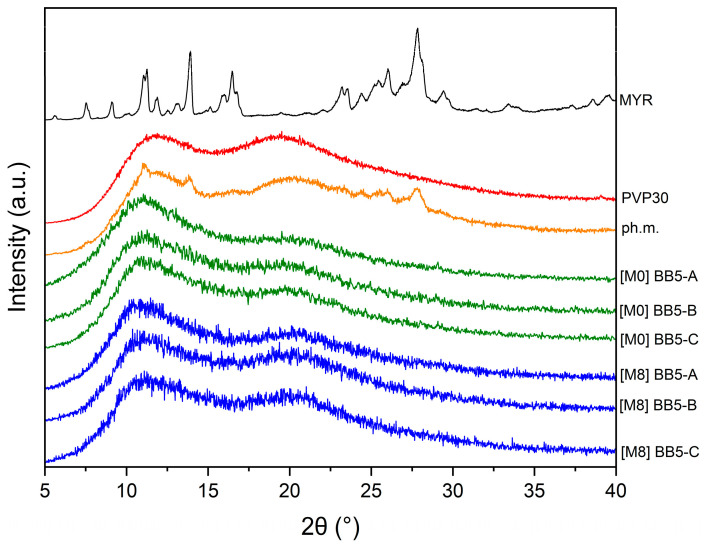
XRPD analysis: stability study. Legend: MYR—myricetin, PVP30—PVP K30, pH.m.—physical mixture, M0—0 month in ambient condition, M8—sample stored for 8 months in ambient conditions.

The XRPD analysis was performed to evaluate the physical stability of the fibers under ambient conditions after 8 months of storage and to determine whether MYR remained in the amorphous state. The diffraction patterns of the stored samples did not exhibit any sharp and well-defined Bragg peaks characteristic of crystalline MYR (Figure 13).

Instead, a broad halo pattern was observed, indicating the absence of long-range molecular order. The diffractograms were comparable to those obtained immediately after fiber preparation, suggesting no detectable recrystallization during storage. This indicates good physical stability of the system and suggests that the polymer matrix effectively inhibited molecular mobility and prevented crystallization over time.

Maintaining the amorphous state is particularly important, as it is typically associated with enhanced apparent solubility and improved dissolution performance. Therefore, the XRPD results demonstrate that the developed fibers provide sufficient stabilization of MYR under the applied storage conditions.

**Figure 14:**
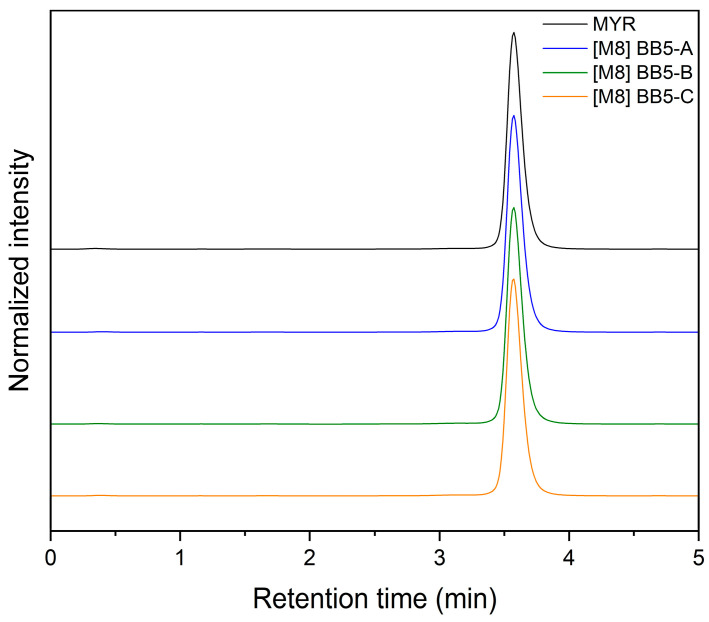
HPLC analysis: stability study. Legend: MYR—myricetin, M8—sample stored for 8 months in ambient conditions.

In addition, HPLC analysis confirmed the absence of detectable degradation products after 8 months of storage. No additional peaks corresponding to degradation products were observed in the chromatograms, and both the retention time and peak purity of MYR remained unchanged (see Figure 14).

These results indicate that MYR retained its chemical stability within the developed fibrous system throughout the storage period. M8—sample stored for 8 months in ambient conditions.

### 2.7. Limitations of the Study

This study has several limitations that should be acknowledged. First, the evaluation of formulation performance was limited to in vitro experiments, and therefore, the results cannot be directly extrapolated to in vivo conditions. Second, the reported apparent solubility values were obtained under non-equilibrium, non-sink conditions and may include contributions from supersaturated molecular species, polymer-associated drug fractions, and/or colloidal aggregates. Consequently, these values do not represent true thermodynamic solubility. Finally, no in vivo validation was performed; therefore, the potential improvement in bioavailability remains hypothetical and requires further pharmacokinetic investigation.

## 3. Materials and Methods

### 3.1. Materials

Myricetin (MYR, ≥98% (HPLC), batch number TGY20211012) was obtained from Xi’an Tian Guangyuan Biotech Co., Ltd. (Xi’an, China). Distilled water was produced using a Direct-Q3 UV water purification system (Merck Millipore, Darmstadt, Germany). Polyvinylpyrrolidone (PVP30), poly(1-vinylpyrrolidone-co-vinyl acetate) (P(VP-co-VAc)), hydroxypropyl-β-cyclodextrin (HP-β-CD), neocuproine, and 2,2-diphenyl-1-picrylhydrazyl (DPPH) were purchased from Sigma-Aldrich (St. Louis, MO, USA). Methanol (MeOH), ethanol (EtOH), and cupric chloride dihydrate were supplied by POCH (Gliwice, Poland). Ammonium acetate (NH_4_Ac) was obtained from Chempur (Piekary Śląskie, Poland).

### 3.2. Viscosity Measurements

The viscosity measurements were performed using a DV2T viscometer (AMETEK Brookfield, Middleboro, MA, USA) equipped with a small-sample adapter. Methanolic stock solutions of the selected carriers (PVP30, HP-β-CD, and P(VP-co-VAc)) were prepared by accurately weighing the appropriate amount of each tested substance and dissolving it in methanol. The solutions were stirred on IKA RCT basic magnetic stirrers (IKA-Werke GmbH & Co. KG, Staufen im Breisgau, Germany) at 400 rpm until complete dissolution was achieved. For PVP30 and HP-β-CD, stock solutions at 30% (*w*/*v*) were prepared; for the copolymer P(VP-co-VAc), a 45% (*w*/*v*) stock solution was used. Subsequently, the stock solutions were diluted to obtain a final concentration of 5% (*w*/*v*). Viscosity measurements were carried out for all prepared solutions. The collected data were used to calculate the specific viscosity ($\eta_{sp}$) according to the following equation [96,97]:

$$\eta_{sp}=\frac{\left(\eta_{in}-\eta_{s}\right)}{\eta_{s}},$$

where $\eta_{sp}$—specific viscosity, $\eta_{in}$—the viscosity of the tested excipient, $\eta_{s}$—the viscosity of the solvent.

The criteria for qualifying polymers for the DOE planned in Section 3.3 included the ability to maintain a stable Taylor cone and a continuous jet without nozzle clogging for at least 60 min under the minimum and maximum process parameters: working distance ($s_{min}$ = 12 cm, $s_{max}$ = 20 cm), applied voltage ($U_{min}$ = 20 kV, $U_{max}$ = 30 kV), and flow rate ($V_{min}$ = 1.5 mL/h, $V_{max}$ = 2.5 mL/h). The following experimental conditions were evaluated:

- s = 12 cm, U = 20 kV, V = 1.5 mL/h
- s = 12 cm, U = 20 kV, V = 2.5 mL/h
- s = 12 cm, U = 30 kV, V = 1.5 mL/h
- s = 12 cm, U = 30 kV, V = 2.5 mL/h
- s = 20 cm, U = 20 kV, V = 1.5 mL/h
- s = 20 cm, U = 20 kV, V = 2.5 mL/h
- s = 20 cm, U = 30 kV, V = 1.5 mL/h
- s = 20 cm, U = 30 kV, V = 2.5 mL/h

### 3.3. Evaluation of Electrospinning Parameters Using a Box–Behnken Experimental Design

To investigate the influence of electrospinning process parameters, a Box–Behnken experimental design with three independent variables and two dependent variables was applied. Based on the literature data [98,99,100,101,102,103], the independent variables selected in this study were the working distance ($s_{min}$ = 12 cm, $s_{max}$ = 20 cm), applied voltage ($U_{min}$ = 20 kV, $U_{max}$ = 30 kV), and flow rate ($V_{min}$ = 1.5 mL/h, $V_{max}$ = 2.5 mL/h). The dependent variables were the apparent aqueous solubility determined at three time points $T_{1}$ = 15 min, $T_{2}$ = 1 h, $T_{3}$ = 24 h. Using Statistica 13.3 software, 15 experimental runs were generated in a randomized order to minimize the risk of systematic errors (Table 4). Experiments 13–15, corresponding to the design’s center point, were used to evaluate the repeatability of the results.

### 3.4. HPLC Analysis

The determination of MYR concentration was carried out using an HPLC–DAD method developed and published by Rosiak et al. [14]. HPLC analyses were performed using a Shimadzu Nexera system (Kyoto, Japan). A ReproSil-Pur Basic-C18 column (5 µm, 100 mm × 4.6 mm; Dr. Maisch GmbH, Ammerbuch, Germany) was used as the stationary phase. The mobile phase consisted of 0.1% formic acid and acetonitrile (60:40, *v*/*v*). Prior to use, the mobile phase was filtered under vacuum through a 0.45 µm nylon membrane filter (Phenomenex, Torrance, CA, USA). The chromatographic conditions were as follows: flow rate, 0.55 mL/min; detection wavelength, 370 nm; column temperature, 30 °C; and injection volume, 2 µL. The method showed linearity described by the equation y = 6.587 × 10^9^x, with a correlation coefficient (R^2^) of 0.999. The repeatability of the process was evaluated at each calibration level, and the relative standard deviation (%RSD) did not exceed 1%, confirming high precision of the analytical procedure. The limits of detection (LOD) and quantification (LOQ) were calculated according to ICH guidelines using the standard deviation of the response (σ) and the slope of the calibration curve (S), based on the formulas LOD = 3.3 σ/S and LOQ = 10 σ/S. The LOD and LOQ values were determined to be 0.000025 mg and 0.000076 mg, respectively.

### 3.5. Apparent Solubility Study

Fragments of the obtained nanofibrous mats were placed in three 10 mL beakers and subsequently dissolved in 4 mL of water maintained at 37 °C. The solutions were stirred using a magnetic stirrer at 150 rpm and 37 °C. At predetermined time points ($T_{1}$ = 15 min, $T_{2}$ = 1 h), 1 mL aliquots were withdrawn and immediately replaced with 1 mL of water. The collected samples were filtered through a 0.22 µm filter into HPLC vials. The MYR concentration was determined using a validated HPLC method [14].

### 3.6. Nanofiber Characterization

#### 3.6.1. SEM Analysis

SEM images were acquired using a Mira-3 Tescan scanning electron microscope (Tescan, Brno, Czech Republic), enabling high-resolution imaging of sample surfaces. The samples were mounted on conductive substrates and coated with a thin carbon layer by sputtering to ensure surface conductivity and prevent electrostatic charging during analysis. Observations were carried out under high-vacuum conditions. The accelerating voltage and magnification were adjusted independently based on the sample characteristics, in accordance with the operating recommendations for the Mira-3 microscope. ImageJ software (version 1.53t, Wayne Rasband and contributors, National Institutes of Health, Bethesda, MD, USA) was used to evaluate the nanofiber size.

#### 3.6.2. XRPD Analysis

X-ray diffraction measurements were carried out on a Bruker D2 Phaser diffractometer (Bruker, Karlsruhe, Germany) using CuKα radiation with a wavelength of 1.54060 Å. The instrument was operated at 30 kV and 10 mA. Data were collected over a 2θ range from 5° to 40°, employing a step size of 0.02° and a counting time of 2 s per step. The diffraction patterns were subsequently processed and evaluated using Origin 2021b software (OriginLab Corporation, Northampton, MA, USA).

#### 3.6.3. FT-IR Analysis

FT-IR measurements were performed using an IRTracer-100 spectrophotometer (Shimadzu, Kyoto, Japan) equipped with a QATR accessory incorporating a diamond ATR crystal. Spectra were recorded in absorbance mode over the range of 400–4000 cm^−1^, with a spectral resolution of 4 cm^−1^ and averaging 400 scans per sample. Data acquisition and subsequent processing, including baseline correction and normalization, were carried out using LabSolution IR software (version 1.86 SP2, Shimadzu, Kyoto, Japan). Interpretation of the results was based on a comparison between the FT-IR spectra of the pure components and those of the prepared nanofibers.

### 3.7. In Vitro Supersaturation and Apparent Solubility Assessment

The in vitro experiments were performed to evaluate the apparent solubility and supersaturation behavior of MYR incorporated into nanofibrous mats (BB5 A–C). The study was conducted under non-sink conditions in an aqueous medium maintained at 37 ± 0.5 °C and continuously stirred at 100 rpm using IKA RCT basic magnetic stirrers (IKA-Werke GmbH & Co. KG, Germany).

Samples of the investigated fibrous mats were placed into beakers containing a defined volume of dissolution medium. At predetermined time intervals, aliquots were withdrawn, filtered, and analyzed by HPLC to determine MYR concentration. The obtained concentration–time data were used to construct supersaturation profiles, which illustrate changes in MYR concentration over time.

### 3.8. Antioxidant Assays

A known amount of BB5 fibers or an excess amount of MYR was weighed and transferred into a 10 mL beaker. Subsequently, 5 mL of water was added, and the mixture was stirred using a magnetic stirrer until the maximum MYR concentration in the system was reached. The stirring time was determined based on the previously established release profile. For the same time interval, an analogous procedure was carried out for crystalline MYR. The concentration of MYR in the aqueous solutions was determined by HPLC analysis.

#### 3.8.1. DPPH Assay

25 µL of the tested sample (aqueous solution of BB5 fibers or MYR) was added to the wells of a 96-well microplate, followed by 175 µL of a methanolic DPPH reagent. The blank sample consisted of 25 µL of water and 175 µL of methanol. The plate was wrapped in aluminum foil, placed in a laboratory incubator, and shaken at 150 rpm at room temperature for 5 min. Subsequently, the plate was incubated in the dark for an additional 25 min without shaking. After the incubation period, absorbance was measured at 517 nm. The degree of DPPH radical scavenging (percentage inhibition, AA%) was calculated according to the following equation [104]:

$$AA\%=\frac{A_{0}-A_{i}}{A_{0}}$$

where $A_{i}$ is the absorbance of the tested sample and $A_{0}$ is the absorbance of the control sample. Based on the obtained data, the IC_50_ value was determined as the concentration of the sample required to neutralize 50% of DPPH free radicals.

#### 3.8.2. CUPRAC Assay

50 µL of the tested sample (aqueous solution of BB5 or MYR) was added to the wells of a 96-well microplate, followed by 150 µL of the CUPRAC reagent. The blank sample consisted of 50 µL of water and 150 µL of the CUPRAC reagent. The plate was wrapped in aluminum foil and incubated in a laboratory incubator with shaking at 150 rpm at room temperature for 30 min. After incubation, absorbance was measured at 450 nm. Based on the collected data, the IC_0.5_ value was determined, defined as the concentration of the sample required to reduce 50% of the available Cu^2+^ ions in the system.

### 3.9. Statistical Analysis

Encapsulation efficiency (EE%) was determined in triplicate (n = 3), and the results are presented as mean ± standard deviation (SD). Statistical analysis of EE% values was performed using one-way analysis of variance (ANOVA) to assess differences among the investigated nanofiber formulations (BB5-A, BB5-B, and BB5-C). Prior to ANOVA, the assumptions of normal distribution and homogeneity of variances were verified. Statistical significance was established at *p* < 0.05. All calculations were performed using Statistica 13.3 (TIBCO Software Inc., Palo Alto, CA, USA).

## 4. Conclusions

An electrospinning-based approach for the fabrication of myricetin-loaded nanofibers using PVP30 was developed, resulting in a substantial enhancement of the apparent aqueous solubility of myricetin. The formation of hydrogen-bonding interactions between the hydroxyl groups of myricetin and the carbonyl groups of PVP30 played a key role in stabilizing myricetin in its amorphous state and improving the physicochemical properties of the nanofibrous systems. SEM analysis confirmed that PVP30 represents a suitable polymeric matrix for electrospinning; however, effective incorporation of MYR requires careful control of dissolution and processing conditions.

The release profile of myricetin from the electrospun nanofibers exhibited a characteristic “spring and parachute” behavior typical of amorphous delivery systems, indicating rapid initial dissolution followed by maintenance of a supersaturated state. The literature suggests that this behavior may enhance the bioavailability of poorly water-soluble compounds. Among the investigated formulations, BB5-A fibers exhibited a great extent of myricetin release and measurable antioxidant activity, as confirmed by DPPH. (IC_50_ = 27 ± 2 μg/mL) and CUPRAC (IC_0.5_ = 120 ± 3 μg/mL) assays.

Overall, the amorphous form of MYR obtained through electrospinning improved its apparent aqueous solubility and enhanced its in vitro antioxidant activity. The results provide insight into the relationship between formulation parameters and nanofiber performance, demonstrating the potential of electrospun systems to enhance the apparent solubility and functional properties of poorly water-soluble compounds. Although no statistically significant optimization effect was identified within the applied experimental design, the study enabled the selection of a formulation exhibiting sustained supersaturation behavior for further characterization. Further investigations are warranted to explore additional process variables, perform more comprehensive morphological and dissolution analyses, and evaluate the in vivo performance of the developed nanofibrous systems.

## Figures and Tables

**Table 1:** Apparent solubility results (myricetin—MYR concentration in mg/mL) obtained for the 15 Box–Behnken experiments (BB) at T_1_ = 15 min and T_2_ = 1 h.

BB	Time [min]	MYR [mg/mL]	BB	Time [min]	MYR [mg/mL]	BB	Time [min]	MYR [mg/mL]
BB1	15	0.07 ± 0.11	BB6	15	0.34 ± 0.16	BB11	15	0.42 ± 0.24
	60	0.54 ± 0.23		60	2.96 ± 0.26		60	2.22 ± 0.11
BB2	15	0.21 ± 0.15	BB7	15	0.49 ± 0.18	BB12	15	0.39 ± 0.07
	60	1.24 ± 0.31		60	2.96 ± 0.13		60	2.22 ± 0.16
BB3	15	0.28 ± 0.12	BB8	15	0.41 ± 0.23	BB13	15	0.45 ± 0.17
	60	1.39 ± 0.15		60	2.09 ± 0.09		60	2.45 ± 0.13
BB4	15	0.48 ± 0.09	BB9	15	0.36 ± 0.04	BB14	15	0.44 ± 0.21
	60	2.57 ± 0.24		60	2.43 ± 0.16		60	2.51 ± 0.35
BB5	15	0.44 ± 0.13	BB10	15	0.46 ± 0.31	BB15	15	0.43 ± 0.09
	60	3.06 ± 0.42		60	2.46 ± 0.18		60	1.73 ± 0.15

**Table 2:** Predicted and actual concentrations of BB5 fibers (A–C) (n = 3).

BB5-A			BB5-B			BB5-C		
Sample	Predicted [mg/mL]	Actual [mg/mL]	Sample	Predicted [mg/mL]	Actual [mg/mL]	Sample	Predicted [mg/mL]	Actual [mg/mL]
1A	0.28	0.18 ± 0.13	1B	0.29	0.20 ± 0.05	1C	0.29	0.22 ± 0.06
2A	0.30	0.25 ± 0.11	2B	0.28	0.19 ± 0.08	2C	0.30	0.24 ± 0.12
3A	0.27	0.16 ± 0.06	3B	0.27	0.17 ± 0.11	3C	0.28	0.23 ± 0.08

**Table 3:** Summary of fiber diameter measurements based on SEM analysis.

Sample	Magnification	Number of Measurements	Mean Diameter [µm]	Minimum Diameter [µm]	Maximum Diameter [µm]
PVP	5000×	50	0.755 ± 0.347	0.215	1.700
	10,000×	50	0.558 ± 0.144	0.290	0.869
BB5 (A)	5000×	50	1.014 ± 0.454	0.319	2.958
	10,000×	50	0.792 ± 0.39	0.340	2.661
BB5 (B)	5000×	50	0.723 ± 0.262	0.240	1.599
	10,000×	50	0.614 ± 0.187	0.215	1.067
BB5 (C)	5000×	50	0.58 ± 0.242	0.152	1.463
	10,000×	50	0.505 ± 0.223	0.109	1.360

**Table 4:** The Box–Behnken design matrix comprising three independent variables (working distance, voltage, and flow rate).

	Distance [cm]	Voltage [kV]	Flow Rate [mL/h]
1.	12.0	20.0	2.0
2.	20.0	20.0	2.0
3.	12.0	30.0	2.0
4.	20.0	30.0	2.0
5.	12.0	25.0	1.5
6.	20.0	25.0	1.5
7.	12.0	25.0	2.5
8.	20.0	25.0	2.5
9.	16.0	20.0	1.5
10.	16.0	30.0	1.5
11.	16.0	20.0	2.5
12.	16.0	30.0	2.5
13.	16.0	25.0	2.0
14.	16.0	25.0	2.0
15.	16.0	25.0	2.0

## Data Availability

The original contributions presented in this study are included in the article and Supplementary Materials. Further inquiries can be directed to the corresponding author.

## Supplementary Materials

The following supporting information can be downloaded at: https://www.mdpi.com/article/10.3390/ph19030449/s1, Table S1: Apparent solubility results (MYR concentration in mg/mL) obtained for the 15 Box–Behnken experiments at T_1_ = 15 min, T_2_ = 1 h oraz T_3_ = 24 h; Figure S1: Fiber diameter distribution of PVP30 nanofibers: Measurement 1—5000× magnification, Measurement 2—10,000× magnification; Figure S2: Fiber diameter distribution of BB5-A nanofibers: Measurement 1—5000× magnification, Measurement 2—10,000× magnification; Figure S3: Fiber diameter distribution of BB5-B nanofibers: Measurement 1—5000× magnification, Measurement 2—10,000× magnification; Figure S4: Fiber diameter distribution of BB5-C nanofibers: Measurement 1—5000× magnification, Measurement 2—10,000× magnification.

## Abbreviations

The following abbreviations are used in this manuscript:

CUPRAC	Cupric ion Reducing Antioxidant Capacity
DPPH	2,2-diphenyl-1-picrylhydrazyl
FT-IR	Fourier-transform infrared spectroscopy
HP-β-CD	hydroxypropyl-β-cyclodextrin
MYR	myricetin
PVP30	polyvinylpyrrolidone K30
P(VP-co-VAc)	poly(1-vinylpyrrolidone-co-vinyl acetate)
SEM	scanning electron microscopy
XRPD	X-ray powder diffraction
