# Supplementary material for: Myricetin Nanofibers as Amorphous Delivery System
Source: Pharmaceuticals (Basel). 2026 Mar 10;19(3):449. doi: 10.3390/ph19030449 (PMC13028992; doi:10.3390/ph19030449)
Supplement: Supplementary file 1 [file pharmaceuticals-19-00449-s001.zip › pharmaceuticals-4116741-supplementary.pdf]

Supplementary Materials

Table S1. Apparent solubility results (MYR concentration in mg/mL) obtained for the 15 Box–Behnken experiments at T_1=15 min, T_2=1 h oraz T_3=24 h

BB	Time [min]	MYR [mg/mL]	BB	Time [min]	MYR [mg/mL]	BB	Time [min]	MYR [mg/mL]
BB1	15	0.35	BB6	15	1.61	BB11	15	1.97
	60	2.52		60	13.92		60	10.46
	1440	6.18		1440	8.36		1440	8.19
BB2	15	0.97	BB7	15	2.32	BB12	15	1.83
	60	5.82		60	13.92		60	10.44
	1440	7.41		1440	5.48		1440	6.86
BB3	15	1.33	BB8	15	1.92	BB13	15	2.14
	60	6.52		60	9.85		60	11.55
	1440	9.63		1440	7.10		1440	6.18
BB4	15	2.26	BB9	15	1.70	BB14	15	2.05
	60	12.09		60	11.43		60	11.79
	1440	7.42		1440	8.37		1440	5.71
BB5	15	2.05	BB10	15	2.18	BB15	15	2.04
	60	14.42		60	11.56		60	8.15
	1440	9.74		1440	4.57		1440	7.17

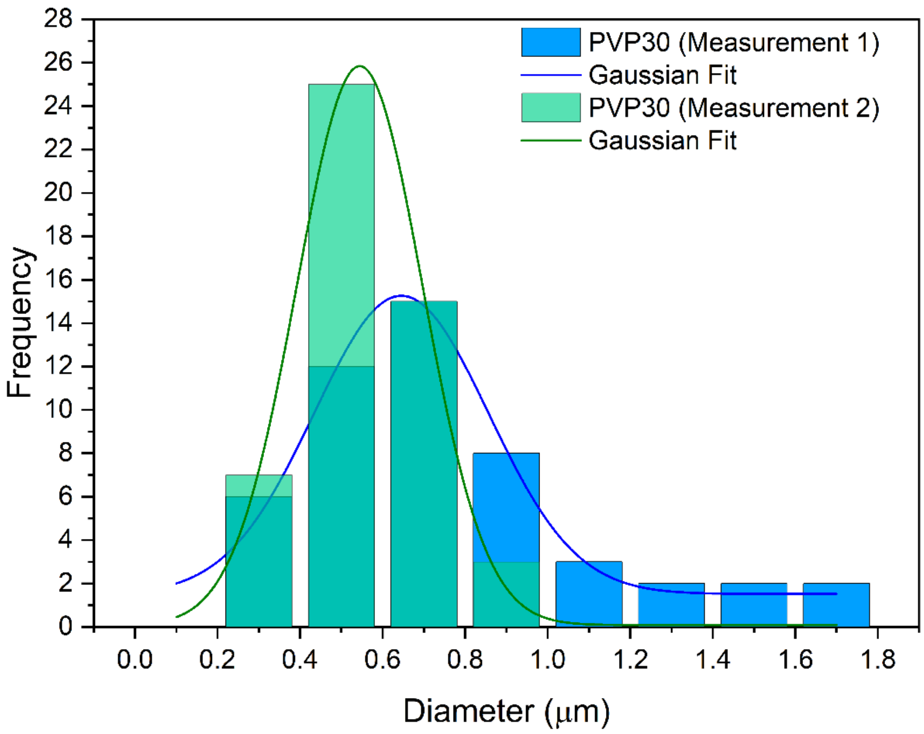

Figure S1. Fiber diameter distribution of PVP30 nanofibers: Measurement 1 – 5000× magnification, Measurement 2 – 10,000× magnification.

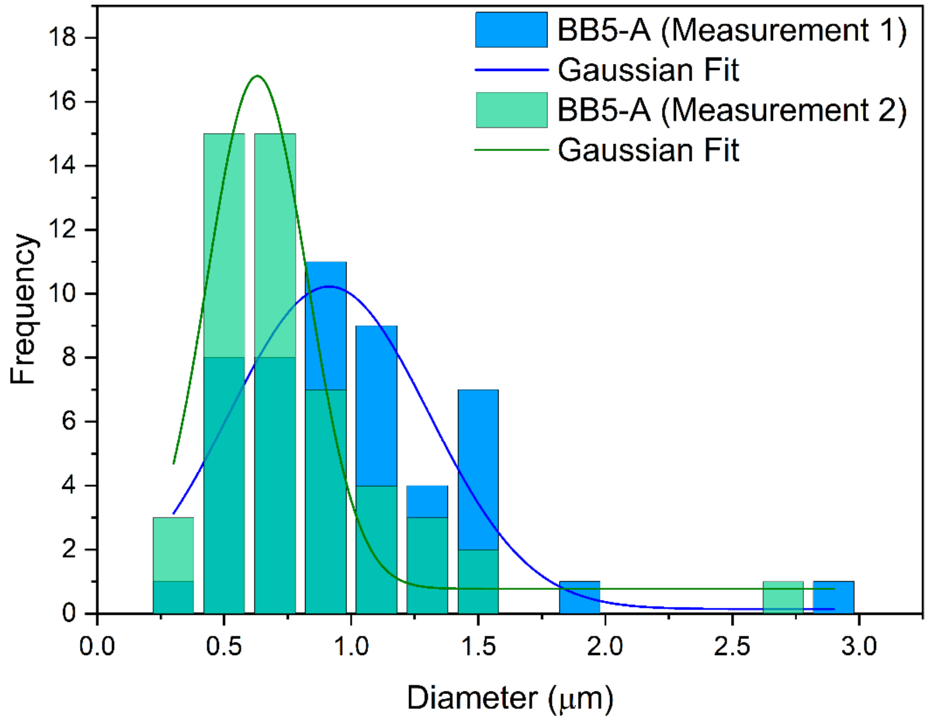

Figure S2. Fiber diameter distribution of BB5-A nanofibers: Measurement 1 – 5000× magnification, Measurement 2 – 10,000× magnification.

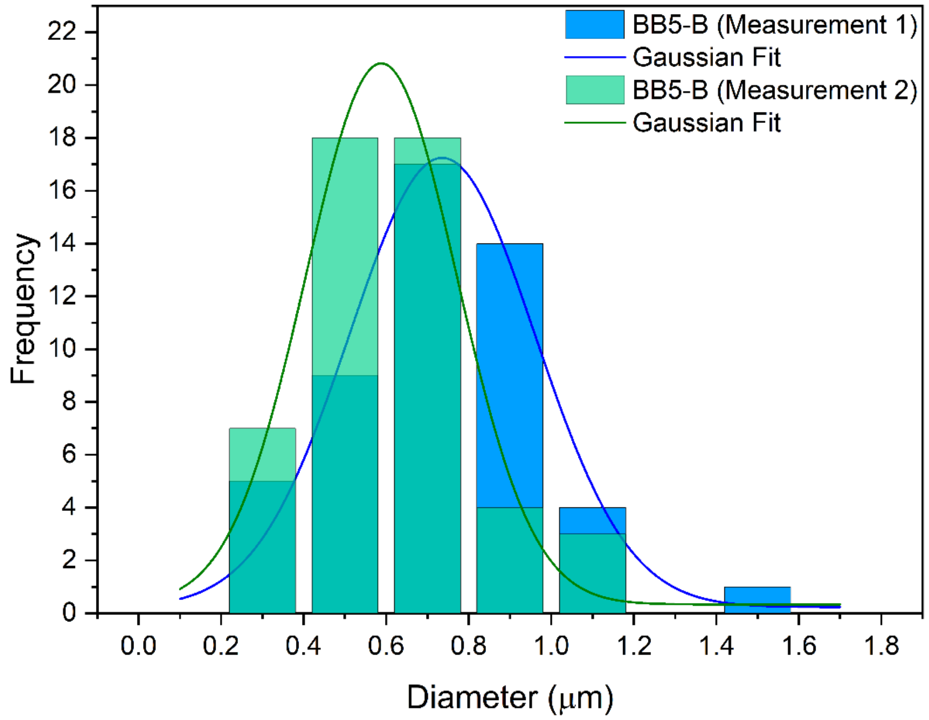

Figure S3. Fiber diameter distribution of BB5-B nanofibers: Measurement 1 – 5000× magnification, Measurement 2 – 10,000× magnification.

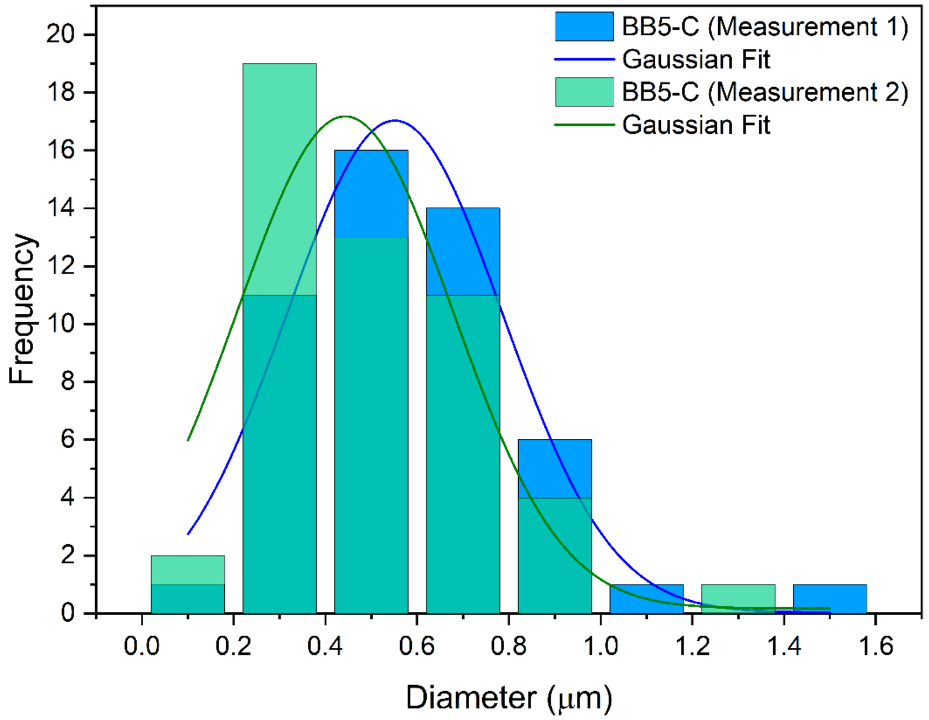

Figure S4. Fiber diameter distribution of BB5-C nanofibers: Measurement 1 – 5000× magnification, Measurement 2 – 10,000× magnification.
